# Supplementary material for: Generating, modeling and evaluating a large-scale set of CRISPR/Cas9 off-target sites with bulges
Source: Nucleic Acids Res. 2024 May 30;52(12):6777–90. doi: 10.1093/nar/gkae428 (PMC11229338; doi:10.1093/nar/gkae428)
Supplement: gkae428_Supplemental_Files [file gkae428_supplemental_files.zip › Supplementary Figures.pdf]

Supplementary Figures

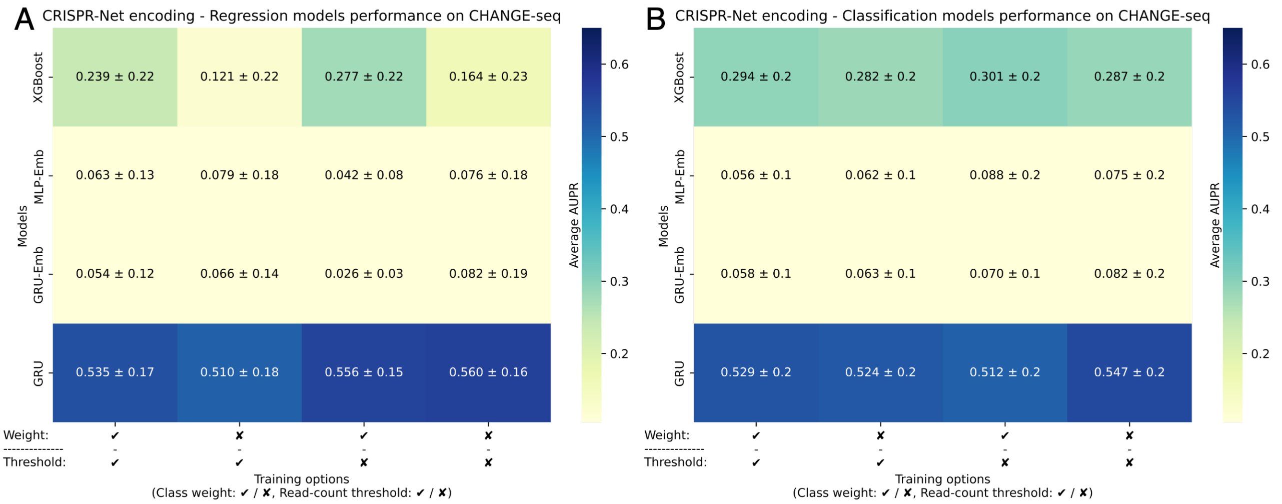

Figure S1. CRISPR-Net encoding prediction performance evaluations on *in vitro* data. (A-B) A comparison of the CH-XGBoost, CH-MLP-Emb, CH-GRU, CH-GRU-Emb regression (A) and classification (B) models trained with CRISPR-Net encoding on the CHANGE-seq data examining the effect of using class weighting and read-count threshold. We gauged the prediction performance of the models by average AUPR over the leave-11-sgRNAs-out cross-validation folds.

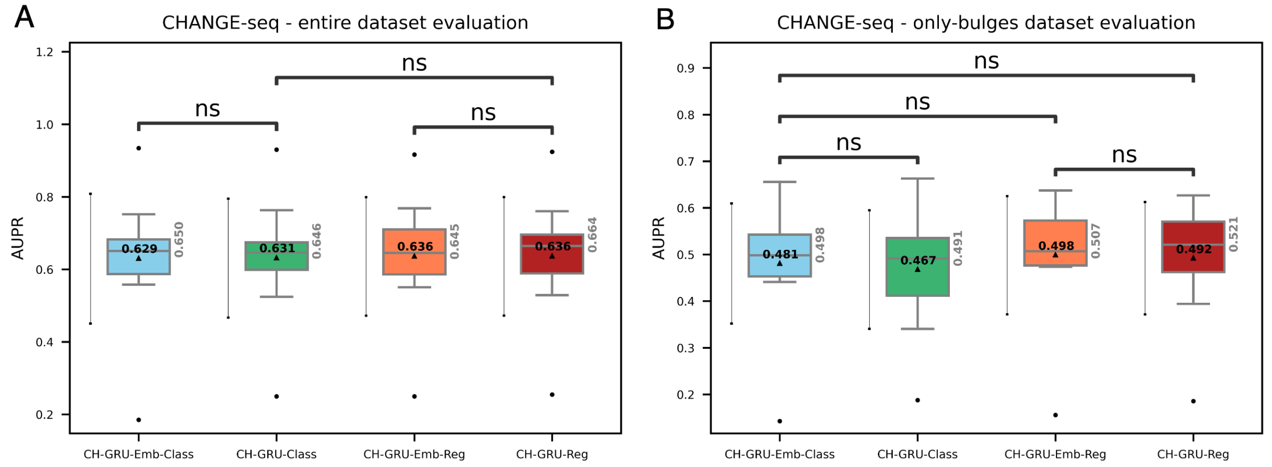

Figure S2. Prediction performance evaluations on *in vitro* data. (A-B) A comparison of the CH-GRU and CH-GRU-Emb regression and classification models' performance on the CHANGE-seq dataset (A) and its only-bulges subset (B). The average AUPR values are denoted by a triangle within each box plot. An error bar of one standard deviation is located on the left of each box plot. The median AUPR values are reported in grey on the right of each box plot. Statistical significance via Wilcoxon signed-rank test is denoted by: ns $5 \cdot 10^{-2} < p \leq 1$.

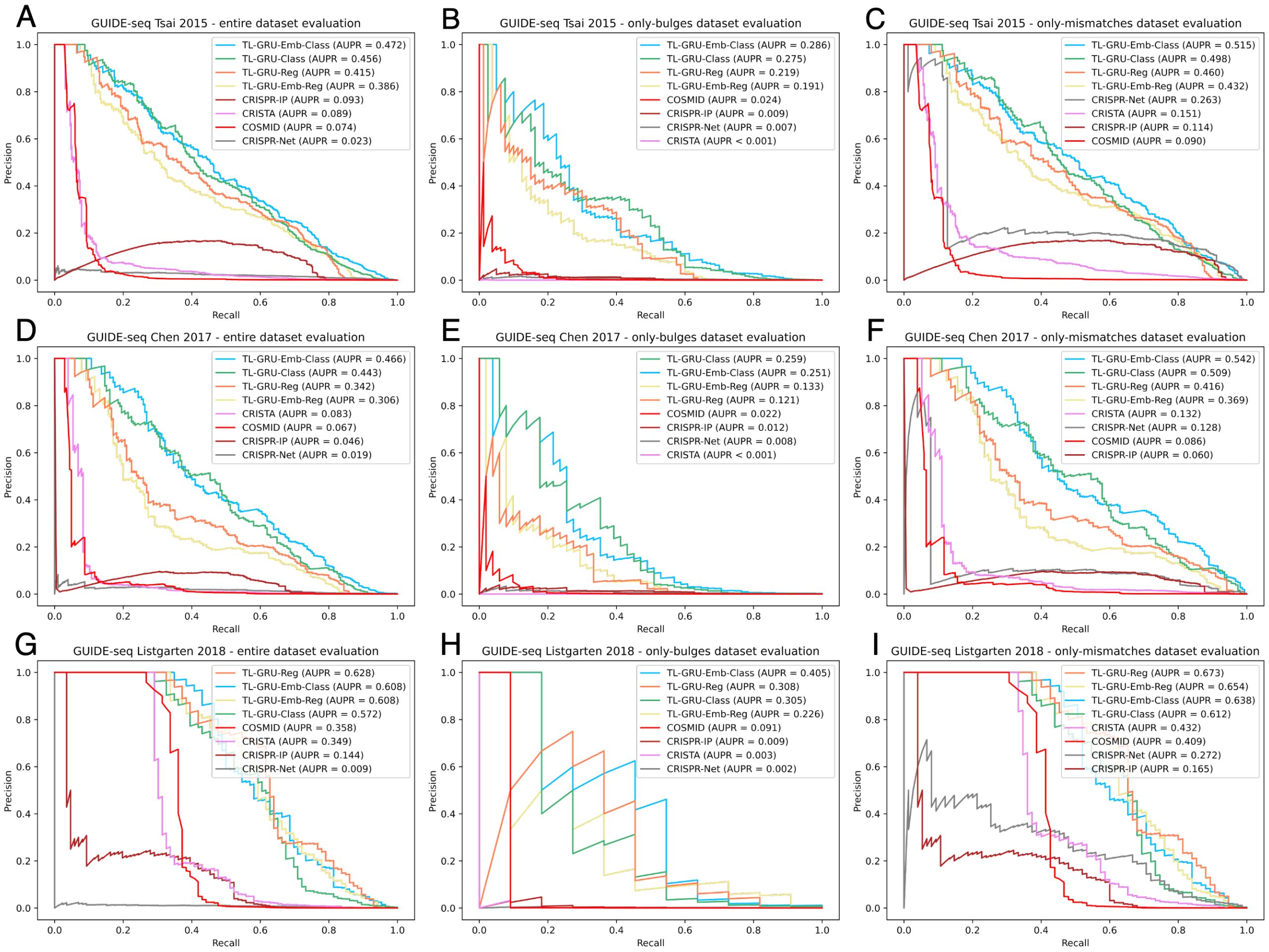

Figure S3. Prediction performance evaluations on independent GUIDE-seq datasets. (A-I) Comparison of our models TL-GRU-Reg, TL-GRU-Emb-Reg, TL-GRU-Class, and TL-GRU-Emb-Class with state-of-the-art methods COSMID, CRISTA, CRISPR-Net, and CRISPR-IP on independent GUIDE-seq datasets: Tsai 2015 (A-C), Chen 2017 (D-F), and Listgarten 2018 (G-I). For each dataset, evaluation is performed on the entire dataset (A, D, and G), its only-bulges subset (B, E, and H), and its only-mismatches subset (C, F, and I).

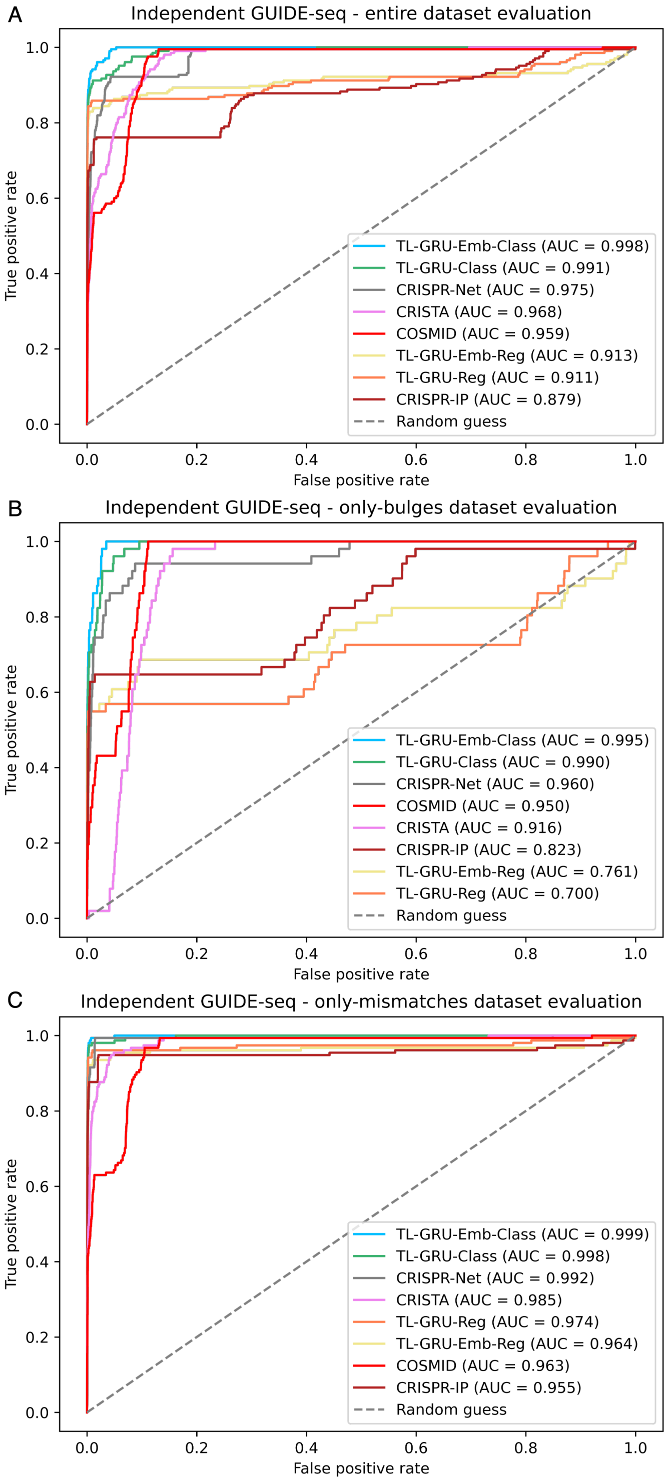

Figure S4. Prediction performance evaluations on the independent GUIDE-seq dataset by the area under the ROC curve. (A-C) Comparison of our models TL-GRU-Reg, TL-GRU-Emb-Reg, TL-GRU-Class, and TL-GRU-Emb-Class with state-of-the-art methods COSMID, CRISTA, CRISPR-Net, and CRISPR-IP on our independent GUIDE-seq benchmark dataset (A), its only-bulges subset (B), and its only-mismatches subset (C).

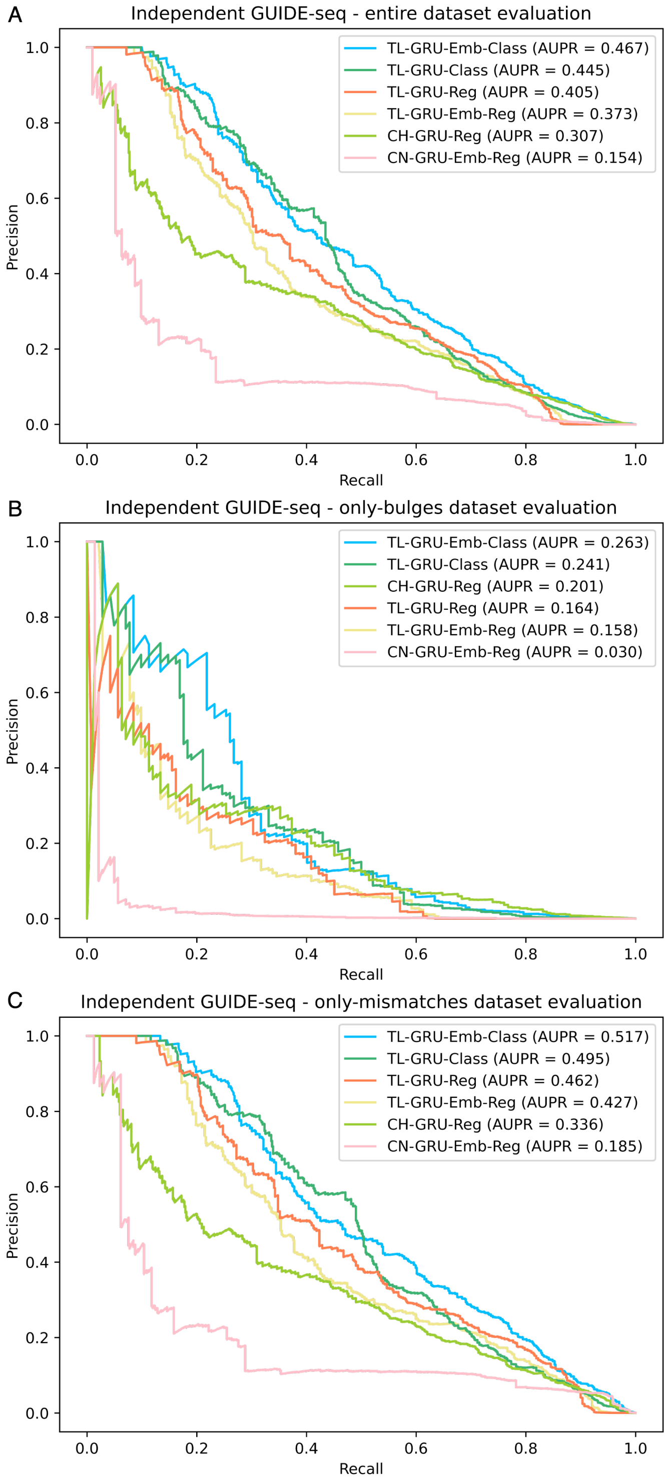

Figure S5. Prediction performance comparison to baseline models on the independent GUIDE-seq dataset. (A-C) Comparison of our models TL-GRU-Reg, TL-GRU-Emb-Reg, TL-GRU-Class, and TL-GRU-Emb-Class with top baseline models trained solely on CHANGE-seq (CH) or CRISPR-Net (CN) datasets, CH-GRU-Reg and CN-GRU-Emb-Reg, on our independent GUIDE-seq benchmark dataset (A), its only-bulges subset (B), and its only-mismatches subset (C). The top baseline models for the CH-GRU and CN-GRU models were picked based on the performance on the entire dataset.

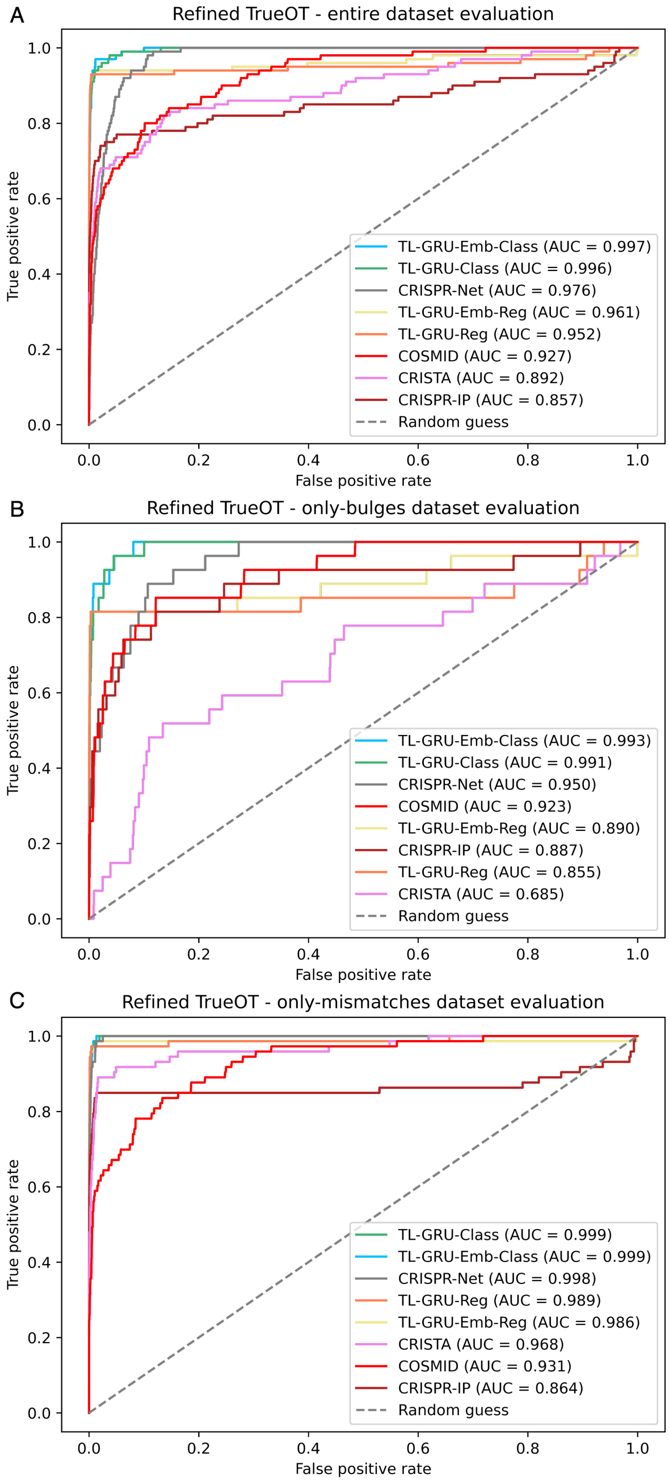

Figure S6. Prediction performance evaluations on the experimentally validated dataset by the area under the ROC curve. (A-C) Comparison of our models TL-GRU-Reg, TL-GRU-Emb-Reg, TL-GRU-Class, and TL-GRU-Emb-Class with state-of-the-art methods COSMID, CRISTA, CRISPR-Net, and CRISPR-IP on our refined TrueOT dataset (A), its only-bulges subset (B), and its only-mismatches subset (C).

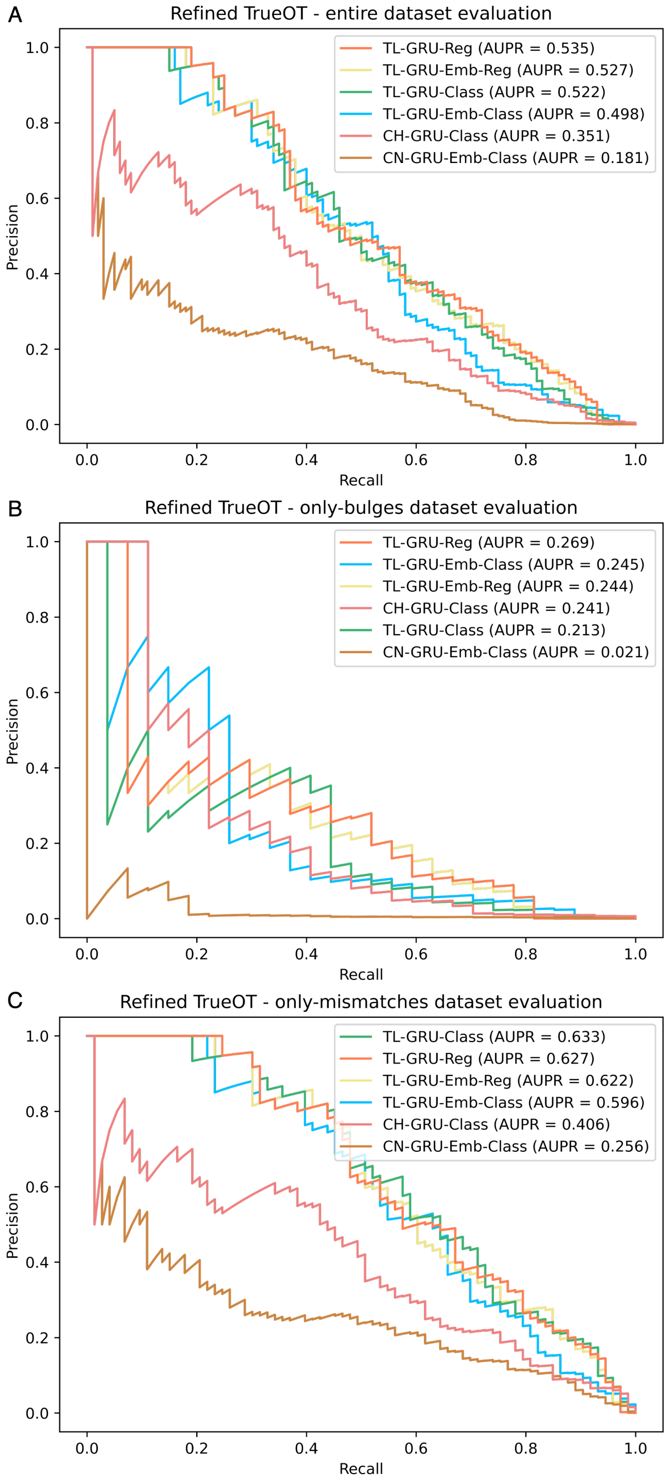

Figure S7. Prediction performance comparison to baseline models on the experimentally validated dataset. (A-C) Comparison of our models TL-GRU-Reg, TL-GRU-Emb-Reg, TL-GRU-Class, and TL-GRU-Emb-Class with top baseline models trained solely on CHANGE-seq (CH) or CRISPR-Net (CN) datasets, CH-GRU-Class and CN-GRU-Emb-Class, on our refined TrueOT benchmark dataset (A), its only-bulges subset (B), and its only-mismatches subset (C). The top baseline models for the CH-GRU and CN-GRU models were picked based on the performance on the entire dataset.

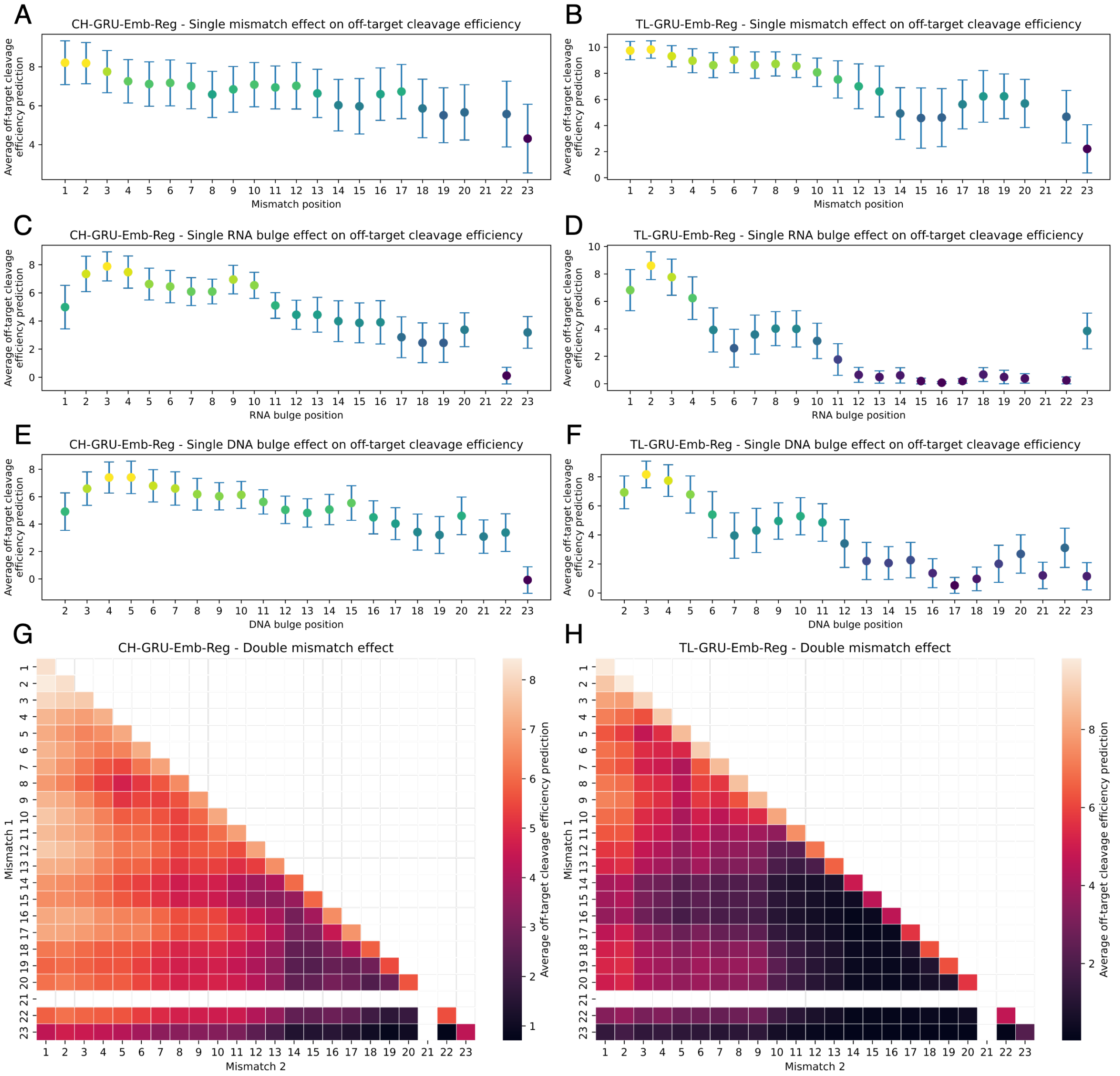

Figure S8. Positional effect of mismatches and bulges on off-target activity learned by our GRU-Emb-Reg model. A-F Positional effect of a single mismatch (A-B), RNA bulge (C-D), or DNA bulge (E-F) compared to the sgRNA, predicted by the CH-GRU-Emb-Reg and TL-GRU-Emb-Reg models for *in vitro* (A, C, and E) and *in cellula* (B, D, and F) data, respectively. Colors correspond to the y-axis values. **G-H** Positional effect of two mismatches compared to the sgRNA predicted by the CH-GRU-Emb-Class (G) and TL-GRU-Emb-Class (H) models.

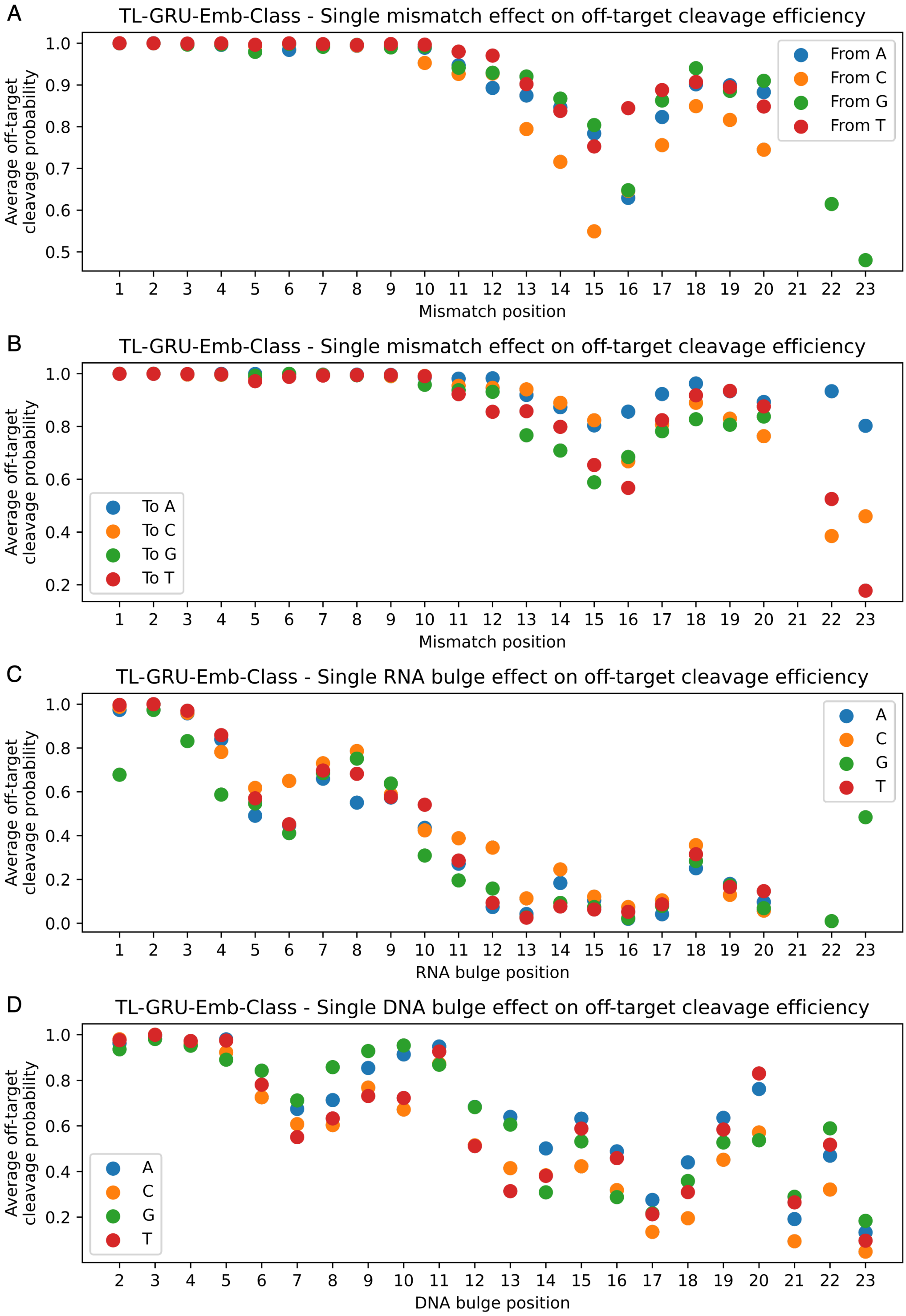

Figure S9. Positional effect of a specific mismatch or DNA/RNA bulge on off-target activity learned by our TL-GRU-Emb-Class model. A-B Positional effect of a specific single mismatch at different positions according to the sgRNA (A) and OTS (B) nucleotide. **C-D** Positional effect of a specific RNA (C) or DNA bulge (D).

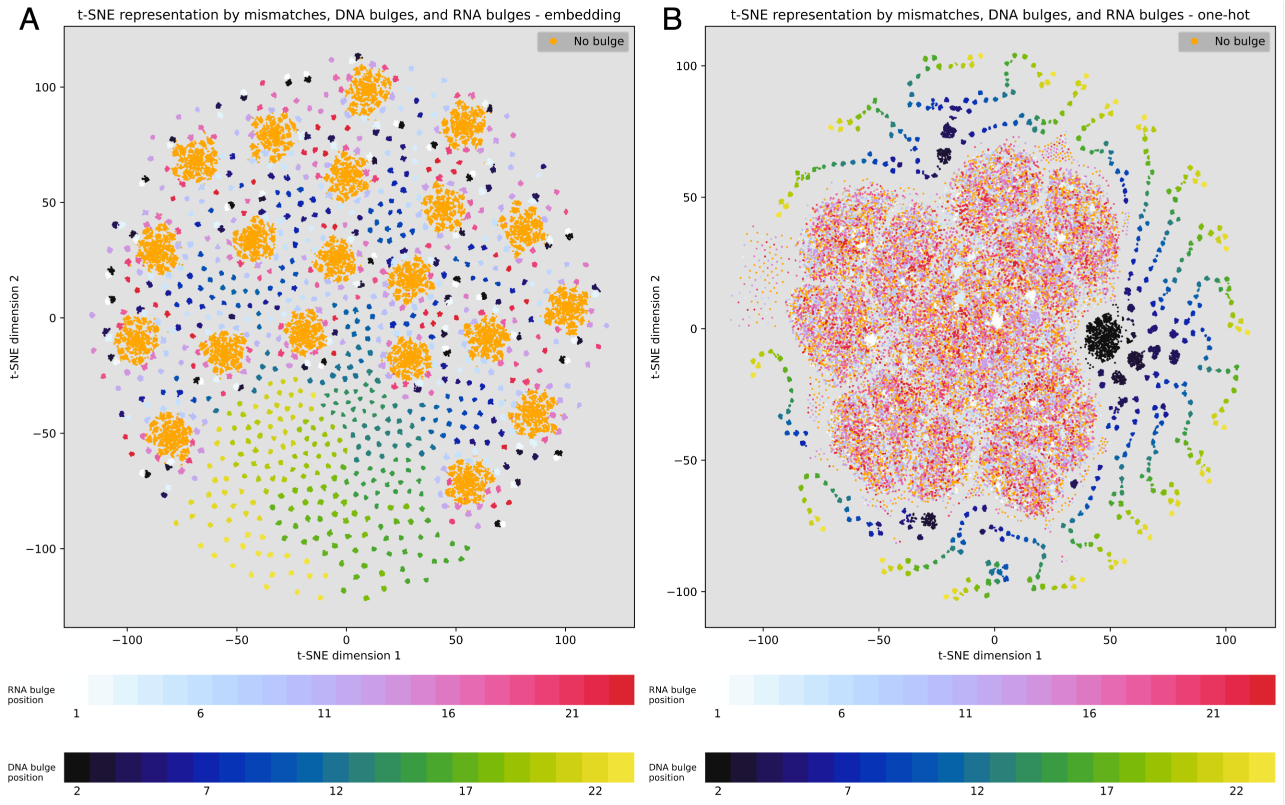

Figure S10. Visualization of the learned embedded representation and one-hot-encoding sequence features of off-target sites with bulges. A Learned embedded representation by the TL-GRU-Emb-Class model projected onto a two-dimensional map using t-SNE without subtracting the sgRNA on-target representations. **B** One-hot-encoding sequence features for a pair of aligned OTS and their sgRNAs projected into a two-dimensional map using t-SNE with subtracting the sgRNA on-target one-hot-encoding representations.
